# The Longitudinal Mediating Effect of Smartphone Dependency on the Relationship between Exercise Time and Subjective Happiness in Adolescents

**DOI:** 10.3390/healthcare11222997

**Published:** 2023-11-20

**Authors:** Inwoo Kim, Hyoyeon Ahn

**Affiliations:** 1 Department of Sports Culture, College of the Arts, Dongguk University, Seoul 04620, Republic of Korea; iwkim@dongguk.edu; 2 Department of Physical Education, College of Education, Seoul National University, Seoul 08826, Republic of Korea

**Keywords:** exercise time, smartphone dependency, subjective happiness, longitudinal mediating effect, panel data

## Abstract

The phenomenon of adolescents engaging in less physical activity as they age raises several concerns. Among these, we hypothesized that this trend may negatively impact their mental health and smartphone dependency. Thus, the aim of this study was to longitudinally examine the mediating effect of smartphone dependency in the relationship between adolescents’ exercise time and subjective well-being. For analysis, publicly available data from the 2018 Korean Children and Youth Panel Survey were utilized, with a total of 2,242 participants’ data included in the analysis. Latent growth modeling results revealed a significant linear decrease in adolescents’ exercise time and subjective well-being each year, while smartphone dependency exhibited an increasing trend. Furthermore, the significance tests of indirect effects indicated that the mediating effect of the changing trend in smartphone dependency between the changing trends in exercise time and subjective happiness in adolescents was statistically significant. These findings suggest that as grade levels increase, reducing exercise time can lead to higher smartphone dependency among adolescents, ultimately resulting in decreased subjective well-being.

## 1. Introduction

The various advantages of appropriate physical activity are widely recognized, leading to a general consensus among most people. In particular, many previous studies emphasize the importance of regular physical activity for adolescents, as this is an important period in forming desirable habits that extend into adulthood [1]. However, although the importance of exercise participation for adolescents’ physical, social, and psychological health is well known, many youths are reported to engage in physical activity below the recommended standards [2]. Furthermore, the prevalence of physical inactivity among adolescents is progressively worsening, leading to ongoing research endeavors and initiatives aimed at enhancing their physical activity levels.

Adolescent physical inactivity is linked to several issues, such as obesity and social deficits. Among these concerns, mental health is widely acknowledged as a critical global issue, and many studies are highlighting the correlation between mental health and physical inactivity in youth. Generally, physical activity and sports participation are known as methods to enhance the mental health of adolescents, with minimal adverse effects [3]. Through relevant research, it has been established that regular participation in physical activity helps adolescents to overcome mental illness, such as depression [4,5] and anxiety [5], while it contributes positively to subjective well-being by enhancing positive affect and life satisfaction [6]. Therefore, given the significant importance of mental health during adolescence, a period characterized by rapid changes and intense stress, efforts to promote their physical activity should also be discussed in connection with mental well-being.

While the scientific mechanisms explaining the positive impact of exercise participation on mental health have not been definitively established, there are existing hypotheses that have been validated through many studies. For example, there are studies suggesting that exercise participation can lead to a reduction in stress hormones or an increase in catecholamine secretion, which may alleviate depressive symptoms [7]. Some research indicates that exercise fulfills basic psychological needs, potentially influencing improvements in quality of life [8]. Despite the accumulation of research findings indicating the potential positive impact of physical activity and exercise participation on the mental health of adolescents, their physical inactivity issue remains unresolved, especially in terms of research focused on the intra-individual decline in physical activity [9,10].

It has been reported that physical activity during adolescence is decreasing annually by an average of 7% across countries [11]. Moreover, in a recent longitudinal study, the number of students engaging in moderate- to vigorous-intensity exercise for at least 60 min a day decreased from 49% before the age of 10 to 14% after the age of 12 [12]. These findings indicate that as adolescents progress through grade levels, their exercise time gradually decreases. Considering the various benefits associated with physical activity, such a decline cannot be overlooked. However, existing research has mainly reported the declining trend in exercise time among adolescents, and empirical studies regarding the impact of such a decline on mental health have not been conducted.

An important point to note is that the subjective well-being or happiness levels of adolescents also decline as they advance to higher grades. According to a study by Twenge, Martin, and Campbell [13], which examined changes in psychological well-being among 8th-, 10th-, and 12th-grade students in the United States from 1991 to 2016, it was found that, since 2012, adolescents have experienced a sudden decrease in self-esteem, life satisfaction, and happiness. This trend is reported to be globally common [14], and, as a result, research on the reasons behind the decline in adolescent happiness levels is also being conducted. A study conducted by Lee et al. [15], which explored the reasons behind the decrease in the happiness levels of adolescents, found that the main reasons were an increase in academic pressure and a reduction in free time with higher grade levels in South Korean middle school students. This signifies that the decline in adolescent happiness levels is a critical global issue, and it also implies the need for research to identify the causes of this decline, such as the reduction in exercise time.

In addition to physical inactivity, another variable that is expected to be associated with the decline in adolescent happiness levels is the level of smartphone dependency. The rapid development of information technology related to smartphones over the past few decades has had a very powerful impact on the lives of modern people. This small device, which brings great convenience and efficiency through numerous functions, such as communication, games, finance, social media, etc., has become a necessity for most people, while side effects due to excessive smartphone use or dependency are also drawing attention as an important social problem [16]. In recent research, it has been noted that there is a global trend of problematic smartphone usage, such as overuse, addiction, and dependence, coinciding with the rapid advancement of relevant technologies and the surge in smartphone utilization [17].

One of the most significant concerns related to this is the mental health of adolescents. According to a related study, the level of psychological distress among adolescents is on the rise, and many studies have provided evidence that this is associated with an increase in smartphone and social media use [18]. Twenge et al. [13] found, from their observational study, that adolescents who spent more time communicating through electronic devices such as smartphones had lower levels of psychological well-being in the United States. Moreover, there is a growing awareness of the excessive dependence on smartphones and its negative impact on mental health. 

Smartphone dependency refers to a psychological state characterized by excessive reliance on smartphones and is known to lead to various negative consequences [19]. This concept is also referred to as problematic smartphone use and is recognized as having negative effects on various aspects, including mental health [16]. According to a study by Yoo [20], smartphone dependency is identified as a significant social issue negatively affecting psychological and behavioral aspects. In addition, it is particularly prominent among adolescents, and there is a growing trend in the number of students excessively reliant on smartphones [21]. A study conducted with Canadian adolescents underscored the negative impact of excessive smartphone use on their mental health [22]. Emphasizing this, the study highlighted the necessity for specific guidelines to mitigate such effects. As previously mentioned, given the declining the levels of happiness and physical activity among adolescents, it is crucial to pay attention to the increasing trend of smartphone dependency, which is known to negatively affect their mental health.

According to the findings of relevant studies, the reduction in exercise time among adolescents and the increase in smartphone dependency both contribute negatively to their mental health. However, the relationship between physical activity and smartphone-related problems has not yet been definitively established. Some studies have suggested that problematic smartphone use may induce inappropriate levels of physical inactivity [23,24]. These studies collectively indicate that adolescents’ excessive smartphone usage can lead to various negative behavioral patterns, with one of them being a decline in physical activity. On the contrary, other studies suggest exercise participation as one of the methods to reduce smartphone dependency. For instance, a study identified that the physical activity levels of university students negatively and directly predicted smartphone dependency [25,26]. Another study suggests that as adolescents engage in more moderate- to vigorous-intensity physical activities, their smartphone dependency may decrease [27]. Given the lack of a clear understanding of the relationship between problematic smartphone usage and physical activity, it is necessary to investigate these two variables for the enhancement of adolescent mental health. In line with this, our study assumes that the decline in physical activity will impact the increase in smartphone dependency.

In summary, while the benefits of physical activity on the mental health of adolescents have been well established over time, the ongoing decline in physical activity time among adolescents remains a significant societal concern. This might be because most relevant studies have focused on elucidating the relationships between two variables, neglecting research into the patterns of change over time and the relationships between such changing trends. In this regard, it is noteworthy that as their age increases, adolescents’ physical activity levels and mental health, including subjective happiness, decrease. Furthermore, it would be valuable to explore the role of smartphone dependency, a factor known to threaten the mental health of adolescents and with a connection to exercise behavior, in the dynamics of these changing patterns. This is also due to the fact that the relationship between exercise behavior and smartphone usage has not been conclusively clarified.

Therefore, the purpose of this study was to longitudinally examine the mediating effect of smartphone dependency in the relationship between adolescent exercise time and subjective happiness. Through the validation of this longitudinal mediation model, useful information about how the changing trends of each variable with increasing age in adolescents may influence one another was obtained. Ultimately, we aimed to provide insights that can contribute to the enhancement of adolescent mental health. For this purpose, we aimed to validate the following hypotheses.

**Hypothesis 1.**  *The exercise time, smartphone dependency, and subjective happiness levels of adolescents will linearly change with age.*

**Hypothesis 2.**  *Changes in adolescents’ exercise time and subjective happiness levels will be mediated by changes in smartphone dependency.*

## 2. Materials and Methods

### 2.1. Data and Participants

We used the secondary dataset of the Korean Child and Youth Panel Survey 2018 (KCYPS 2018) collected by the National Youth Policy Institute (NYPI) in Korea. KCYPS 2018 is an annual follow-up survey on the growth and development of all 4th-grade elementary school children and 1st-year middle school adolescents nationwide, using a stratified cluster sampling method based on residential areas (e.g., major cities, small towns, villages). Schools were then randomly selected in each residential area, and participation in the survey was voluntary. Data were collected using the Tablet-Assisted Personal Interview (TAPI) method to address the limitations of other previous panel surveys, such as existing input errors and time-consuming data collection. The data and codebook are publicly available on the NYPI website: https://www.nypi.re.kr/archive/mps (accessed on 1 July 2023) for anyone to access, and they can be freely downloaded after obtaining consent for data usage.

This study used the dataset from the first to the third wave of surveys conducted on 1st-year middle school students starting from 2018. The analysis was conducted using data from 2242 participants who responded to all items without any missing values for all three years, and there were 1204 males and 1038 females.

### 2.2. Measures

#### 2.2.1. Exercise Hours

In the KCYPS 2018, adolescent exercise time was measured using a single item, and we used the item in this study for analysis. This item was adapted from the International Physical Activity Questionnaire (IPAQ) [28], which measures moderate-intensity exercise time (“The hours you spent on exercise that made you sweat in the past week”). It was measured on a 5-point Likert scale (1 = none, 2 = 1 h, 3 = 2 h, 4 = 3 h, 5 = 4 h or more).

#### 2.2.2. Smartphone Dependency

To measure the smartphone dependency of the participants, the Smartphone Addiction Proneness Scale (SAPS) was used in the KCYPS 2018 [29]. The SAPS consists of a total of 15 items divided into four subscales: disturbance of adaptive functions (5 items), virtual world orientation (2 items), withdrawal (4 items), and tolerance (4 items). The items were modified for adolescents and measured using a 4-point Likert scale (1 = strongly disagree, 2 = disagree, 3 = agree, 4 = strongly agree). Examples of the questions for each factor included “I have had moments where I couldn’t concentrate on what I was doing (studying) because I was using my smartphone” (disturbance of adaptive functions); “when I cannot use a smartphone, I feel like I have lost the entire world” (virtual world orientation); “If I couldn’t use my smartphone, it would be difficult to endure” (withdrawal); “I try cutting my smartphone usage time, but I fail” (tolerance). We used the mean scores of the overall responses for the analysis, and the Cronbach’s alpha coefficients in this study were 0.876 (wave 1), 0.868 (wave 2), and 0.877 (wave 3).

#### 2.2.3. Subjective Happiness

In the KCYPS 2018, subjective happiness was measured using the Happiness Index Scale developed and validated by Lee et al. [30]. This scale consists of four items, including “I am a very happy person overall,” rated on a 4-point Likert (1 = strongly disagree, 2 = disagree, 3 = agree, 4 = strongly agree). Higher mean scores indicate higher levels of happiness, and the Cronbach’s alpha coefficients in this study were 0.796 (wave 1), 0.763 (wave 2), and 0.730 (wave 3).

### 2.3. Analysis

In this study, we conducted descriptive statistics and correlation analysis using IBM SPSS Statistics 26. In addition, to validate the longitudinal mediating model, we performed latent growth modeling (LGM) analysis using the AMOS 21.0 program. Latent growth modeling analysis is a technique that allows for both cross-sectional analysis through the examination of relationships between initial values and the analysis of relationships between the rates of change of each variable simultaneously. 

The first step of the LGM analysis involved verifying whether the patterns of change in each variable were linear each year. This process compared the fit indices of models assuming that students’ exercise time, smartphone dependency, and subjective happiness levels would linearly change with each grade against models assuming no such linear change. The subsequent step validated a research model examining the mediating effects between initial values of each variable and mediating effects between their slopes. Through this step, we could understand not only the relationships between a teenager’s exercise time, smartphone dependency, and subjective happiness but also how the changes in these three variables over time might influence each other. Finally, to validate the significance of these mediating effects, we employed the bootstrapping method for X^2^ difference test and significance test of indirect effects. The confidence interval was set to 95%, and the significance level (α) was set to 0.05.

## 3. Results

### 3.1. Descriptive Statistical Analysis

In the descriptive statistical analysis, we examined the mean (M), standard deviation (SD), skewness, and kurtosis of each variable for each year (w1~w3), and the results can be found in Table 1. Results revealed that the average exercise time, which was 3.03 at the initial wave of middle school 1st grade, gradually decreased over time to 2.32 at wave 3. In contrast, smartphone dependency showed an increasing trend from 2.04 at the onset of middle school 1st grade to 2.18 at the third grade. Additionally, the subjective happiness level, initially at 3.13 during wave 1, displayed a decreasing trend as students progressed to 3.06 at the highest grade. The verification of normality was conducted by checking skewness (≤2) and kurtosis (≤4.0). The results indicated that all variables met the criteria, confirming their normality.

The correlation analysis involved deriving the correlation coefficients for the mean values of the three variables for each year (w1~w3). Results revealed significant relationships among all three variables (Table 2). In particular, exercise time and subjective happiness were positively correlated, while smartphone dependency exhibited negative correlations with the other two variables.

### 3.2. Latent Growth Modeling Analysis

Through latent growth modeling analysis, we examined whether the changes in exercise time, smartphone dependency, and subjective happiness as participants’ age increased were linear and statistically significant. Table 3 indicates that all three variables showed a good fit for the linear model, and the overall fit of the research model was also satisfactory. This means that participants’ exercise time and subjective happiness decreased linearly as they grew older, while smartphone dependency increased every year. 

**Figure 1:**
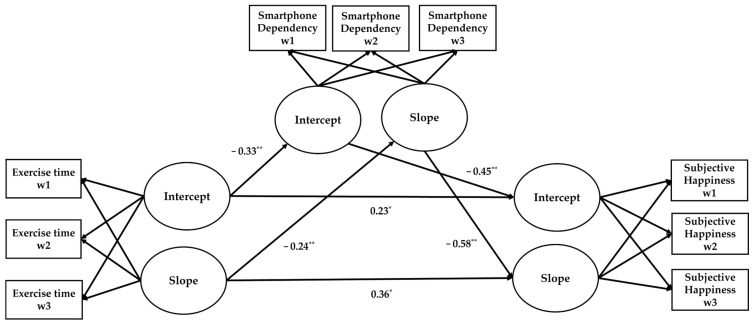
Research model (** *p* < 0.01, * *p* < 0.05), intercept: initial value, slope: change rate, w1 = wave 1, w2 = wave 2, w3 = wave 3.

The research model that we established to verify the longitudinal mediation effects is shown in Figure 1. It was identified that the initial value of exercise time negatively predicted the initial value of smartphone dependency (β = −0.33, *p* < 0.01) and positively predicted the initial value of subjective happiness (β = 0.23, *p* < 0.05). Additionally, the initial value of smartphone dependency negatively predicted the initial value of subjective happiness (β = −0.45, *p* < 0.01). The relationships between the change rates of each variable were all found to be significant. The change rate of exercise time negatively predicted the change rate of smartphone dependency (β = −0.24, *p* < 0.01) but positively predicted the change rate of subjective happiness (β = −0.36, *p* < 0.05). Similarly, the change rate of smartphone dependency negatively predicted the change rate of subjective happiness (β = −0.58, *p* < 0.01).

### 3.3. Mediating Effect

An indirect effect analysis through the bootstrapping method was performed in the 95% confidence interval to verify the mediating effect of the research model. The indirect effect of exercise time on subjective happiness through smartphone dependency was found to be significant for both the paths between the initial values (β = 0.145, *p <* 0.01) of each variable and the paths between the change rates (β = 0.141, *p <* 0.01) of each variable (Table 4).

## 4. Discussion

Despite the widely recognized positive influence of physical activity and exercise participation on adolescent mental health, many students fail to engage in a sufficient level of physical activity. Consequently, both their physical inactivity and low levels of mental well-being persist as significant societal concerns. Furthermore, what demands even greater attention is the fact that adolescents’ levels of physical activity and mental health deteriorate as they progress through their school years. While numerous studies have accumulated regarding the relationship between exercise and mental health, there has been limited research into the dynamics of these trends over time. Additionally, the recent rise of smartphone dependency as a pressing social issue, coinciding with increasing age among adolescents, adds a layer of complexity. Smartphone dependency is identified as a detrimental factor for adolescent mental health, and exercise is suggested as a potential means to alleviate this dependency.

In essence, we postulate that participation in physical activity can contribute to lowering smartphone dependency, subsequently promoting the mental well-being of adolescents. Specifically, this study aimed to investigate the intrapersonal changes in exercise time, subjective well-being, and smartphone dependency that occur with increasing age among adolescents. To explore these changing trends and their interrelationships, we utilized LGM analysis with panel data collected longitudinally.

The results of the latent growth modeling analysis indicate that Korean adolescents experience a decline in exercise time from the 1st to the 3rd year of middle school. This decline aligns with previous research findings [9,10,11,12] and holds significance since it highlights the importance of emphasizing the potential risks associated with this trend. In particular, the decrease in physical activity among adolescents with increasing age can have negative implications for health-related indicators in adulthood [11]. Therefore, promoting exercise participation during early adolescence is crucial for overall well-being. Additionally, shedding light on the negative impacts of progressively reduced physical activity as students advance through school would provide a rationale for efforts to mitigate this declining trend.

Furthermore, the subjective happiness levels of participants exhibited a gradual decrease as they progressed through middle school. While the decline was not substantial, it was in accordance with prior studies and serves as an indicator warranting further investigation [13,14]. This suggests that while discussing the severity of the decline in happiness levels among Korean adolescents may not be appropriate, examining the reasons behind this trend in relation to age is essential. The findings from previous studies, indicating the universality of this phenomenon worldwide [14], and its association with excessive smartphone use due to technological advancements [13], provide justification for an examination of the impact of smartphone dependency in this study.

Notably, smartphone dependency displayed an increasing trend with age. The escalating smartphone usage and dependency among adolescents are causing significant global concern due to their adverse effects on psychological and behavioral aspects [16,17,18,19,20,21]. The age-related increase in smartphone dependency, consistent with previous research findings [21], underscores the need to explore the factors associated with this increase. In previous research, it has been suggested that the increase in smartphone dependency among early adolescents could be attributed to rising stress factors, such as pressure or anxiety, leading individuals to rely more on smartphones as a means of alleviating these stresses [21]. In this study, we hypothesized that the increasing trend in smartphone dependency may be related to the declining trend in exercise, partly because exercise is a prominent means of stress relief.

The key findings of our study reveal a relationship between changing trends in these three variables. The analysis of the research model validates that a decrease in exercise time negatively predicts an increase in smartphone dependency, while positively predicting subjective well-being. As anticipated, smartphone dependency negatively predicts subjective well-being. Furthermore, the results indicate a significant mediating effect of smartphone dependency on the relationships between exercise time and subjective well-being, both in terms of initial values and change rates.

In conclusion, the direct impact of decreasing exercise time on reduced happiness levels emphasizes the need for heightened awareness regarding the phenomenon of adolescents’ reduced exercise time as they progress through school. This is consistent with various related research findings, such as a review study confirming that the physical activity of adolescents and young adults can enhance not only their physical health but also their mental and social well-being [31], or research results indicating a positive correlation between the frequency of physical activity participation among adolescents and their subjective happiness [32]. While the positive effects of physical activity on mental health are well established, they have yet to be used to effectively counteract the growing problem of adolescent physical inactivity. Therefore, given the results of present study, it may be more effective to focus on examining the direct consequences of reduced exercise time, such as reducing exercise time to accommodate academic demands.

Additionally, the mediating effect of smartphone dependency, as revealed in our study, highlights the potential to use exercise as a method to reduce this dependency. While numerous studies emphasize the detrimental effects of increasing smartphone dependency on adolescent mental health [13,16,18], emerging research suggests that physical activity may be employed to mitigate this dependency [25,27]. In particular, this aligns with research results indicating that aerobic exercise interventions reduced the desire for mobile phone use among university students with high mobile phone dependency [33] and identifying that exercise negatively predicted smartphone addiction [34]. Our study provides support for this notion; furthermore, the findings underscore the severity of the decline in exercise time with increasing age, which in turn leads to an increase in smartphone dependency, negatively affecting happiness levels among adolescents.

## 5. Conclusions

### 5.1. General Conclusions

We aimed to longitudinally examine the mediating effect of smartphone dependency in the relationship between adolescent exercise time and subjective well-being. To achieve this objective, latent growth modeling analysis was conducted using panel data. The study revealed that as adolescents age, the decrease in exercise time can directly reduce subjective happiness levels and indirectly elevate smartphone dependency.

This study contributes valuable insights into the intricate interplay between exercise, smartphone dependency, and subjective happiness among adolescents. It calls for comprehensive efforts to address the multifaceted issues surrounding adolescent physical activity and mental health, taking into account the changing trends observed in these variables. Specifically, based on the results of this study, it can be emphasized that there is a need for institutional and educational efforts to prevent a decrease in weekly exercise time among middle school students as they progress through school. In the absence of such efforts, it can be added that students’ smartphone dependency is likely to increase, leading to a gradual decline in their levels of happiness. In the same context, it could be proposed to increase the time spent in physical activity as a measure to reduce smartphone dependency among adolescents and promote their mental well-being.

### 5.2. Limitations

The limitations of this study and suggestions are as follows. Firstly, it is important to note that the data used in this study were collected from Korean middle school students. Therefore, caution should be exercised when applying the results of this study to different cultural contexts. Secondly, both smartphone dependency and subjective well-being in adolescents are multifaceted concepts influenced by numerous latent variables. Despite their complexity, this study was unable to account for all potential factors influencing these variables. This is a common limitation in studies utilizing panel data as the researcher’s intent or purpose may not be fully reflected in the data collection process. Thirdly, this study did not consider important control variables that could influence the research results, such as gender and the amount of smartphone usage. Exercise behavior and smartphone usage patterns among adolescents may vary depending on gender, and smartphone dependency is likely to be influenced by factors like screen time.

### 5.3. Future Prospects

The above limitations should be considered when interpreting the results of this study or planning future research. Researchers should take into account diverse cultural contexts and explore other variables that may mediate the relationship between adolescents’ exercise time and happiness, aside from smartphone dependency. Additionally, exploring control variables such as gender based on a literature review, focusing on factors influencing adolescents’ exercise time, smartphone usage, and happiness levels and incorporating them into the analysis, could offer more substantive insights into the effectiveness of exercise in reducing smartphone dependency and enhancing happiness among adolescents.

## Figures and Tables

**Table 1:** Descriptive statistics.

Variables	M	SD	Skewness	Kurtosis
exercise time w1	3.03	1.45	0.06	−1.37
exercise time w2	2.81	1.40	0.25	−1.18
exercise time w3	2.32	1.38	0.77	−0.66
smartphone dependency w1	2.04	0.49	0.21	−0.07
smartphone dependency w2	2.13	0.47	−0.07	−0.10
smartphone dependency w3	2.18	0.49	−0.10	−0.32
subjective happiness w1	3.13	0.55	−0.33	0.31
subjective happiness w2	3.07	0.48	0.03	0.45
subjective happiness w3	3.06	0.45	−0.09	1.01

Note: w1 = wave 1, w2 = wave 2, w3 = wave 3.

**Table 2:** Correlation matrix.

Variables	1.	2.	3.	4.	5.	6.	7.	8.
1. exercise time w1	1							
2. exercise time w2	0.427 **	1						
3. exercise time w3	0.314 **	0.337 **	1					
4. smartphone dependency w1	−0.176 **	−0.144 **	−0.110 **	1				
5. smartphone dependency w2	−0.109 **	−0.172 **	−0.101 **	0.447 **	1			
6. smartphone dependency w3	−0.128 **	−0.105 **	−0.151 **	0.360 **	0.481 **	1		
7. subjective happiness w1	0.168 **	0.164 **	0.088 **	−0.326 **	−0.218 **	−0.177 **	1	
8. subjective happiness w2	0.106 **	0.216 **	0.096 **	−0.166 **	−0.324 **	−0.176 **	0.437 **	1
9.subjective happiness w3	0.101 **	0.129 **	0.149 **	−0.167 **	−0.186 **	−0.261 **	0.368 **	0.457 **

Note: ** *p* < 0.01, w1 = wave 1, w2 = wave 2, w3 = wave 3.

**Table 3:** Comparison between the no change models and the linear change models.

Variables	Models	χ^2^	df	RMSEA	NFI	TLI	CFI	SRMR
Exercise Time	no change	469.423 **	6	0.186	0.422	0.714	0.427	0.424
	linear	28.917 **	3	0.062	0.964	0.968	0.968	0.013
Smartphone Dependency	no change	227.464 **	6	0.102	0.805	0.905	0.809	0.038
	linear	7.025	3	0.024	0.994	0.997	0.997	0.007
Subjective Happiness	no change	187.813 **	6	0.116	0.83	0.917	0.835	0.028
	linear	41.479 **	3	0.081	0.957	0.96	0.96	0.030
Research Model		260.895 **	24	0.066	0.933	0.907	0.938	0.029

Note: ** *p* < 0.01.

**Table 4:** Direct, indirect, and total effect.

	Path	Direct Effect	Indirect Effect	Total Effect
	Exercise Time → Subjective Happiness	0.227 **	0.145**	0.373 **
Intercept	Exercise Time → Smartphone Dependency	−0.334 **		−0.334 **
	Smartphone Dependency → Subjective Happiness	−0.435 **		−0.435 **
	Exercise Time → Subjective Happiness	0.364 **	0.141 **	−0.505 **
Slope	Exercise Time → Smartphone Dependency	−0.243 **		−0.243 **
	Smartphone Dependency → Subjective Happiness	−0.583 **		−0.583 **

Note: ** *p* < 0.01

## Data Availability

The data presented in this study are available on request from the corresponding author.
